# Survey of sodium gluconate content in retail Cheddar cheese

**DOI:** 10.3168/jdsc.2024-0569

**Published:** 2024-09-02

**Authors:** N. Mishra, D.J. McMahon, C.J. Oberg, T.S. Oberg

**Affiliations:** 1 Western Dairy Center, Utah State University, Logan, UT 84322; 2 Department of Microbiology, Weber State University, Ogden, UT 84408

## Abstract

The presence of sodium gluconate in Cheddar cheese, which can be added as a processing aid during manufacture, has been shown as a contributing factor to gas production and gas defects in aged cheese. A survey of gluconate content in retail Cheddar cheese was performed. Fifty-three retail packs of Cheddar cheese were purchased from stores in Utah as blocks, shreds, sliced cheeses, or individual-sized cuts and sticks. This included mild, medium, and sharp cheeses. Analysis of gluconate content was performed using an enzyme assay. Levels of gluconate ≥0.1% were found in 7 samples with the highest level being 0.45% (wt/wt).

## Highlights

• Some Cheddar cheese contains gluconate.
• Sodium gluconate can be added as a processing aid to prevent lactate crystal formation in cheese.
• Gluconate levels in cheese ranged from none to 0.45%.

## Graphical Abstract

**Figure:**
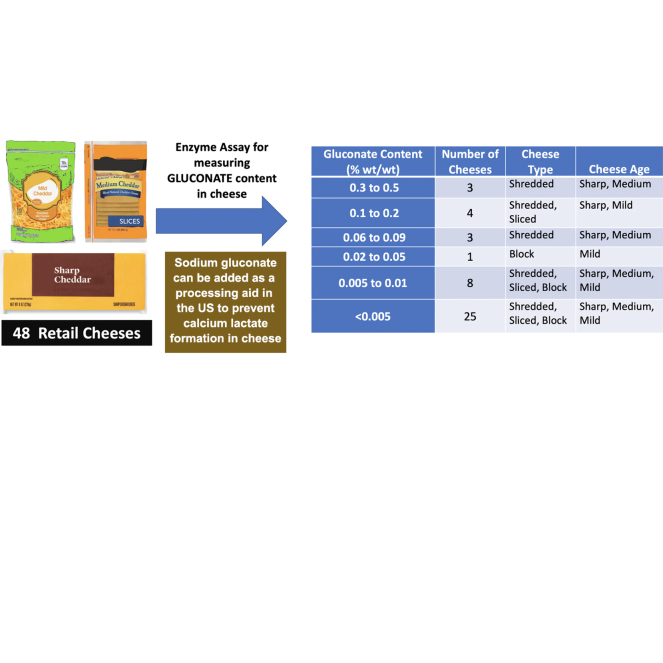
**Summary:** Sodium gluconate can be added to Cheddar cheese as a processing aid to decrease the formation of calcium lactate during aging, but the addition of sodium gluconate to Cheddar cheese has been shown to increase the potential for late gas defects in aged Cheddar cheese. This study measured the concentration of sodium gluconate in retail Cheddar cheese samples using an enzymatic analysis kit for sodium gluconate. Of the 48 retail cheese samples, 3 had levels that indicate the intentional addition of sodium gluconate as a processing aid.

*Paucilactobacillus wasatchensis* is a nonstarter lactic acid bacteria (**NSLAB**) that was originally isolated from a gassy cheese. After initially being assigned to the *Lactobacillus* genus (Oberg et al., 2016), when the lactobacilli were divided into 27 different genus, it was grouped together with other lactobacilli that specialize in utilizing limited substrates for growth into the *Paucilactobacillus* genus (Oberg et al., 2022). Isolation and enumeration of *Pa. wasatchensis* from cheese has been difficult in the presence of higher numbers of other faster growing NSLAB, such as *Latilactobacillus curvatus.* It can be enumerated using a 2-step process by growing on carbohydrate-restricted de Man, Rogosa, and Sharpe (MRS) agar in which ribose is the only added sugar (Ortakci et al., 2015a). *Paucilactobacillus wasatchensis* uses ribose by the pentose phosphate pathway, and can also use galactose by converting it into a 5-carbon substrate via the Leloir pathway, which liberates a carbon dioxide molecule (Ortakci et al., 2015a). When there is galactose in the cheese, there can be unwanted gas production during cheese storage (Ortakci et al., 2015b) such as can occur when *Streptococcus thermophilus* is used as a starter culture in Cheddar cheese (Ortakci et al., 2015c). It has recently been observed that *Pa. wasatchensis* can also use gluconate as a 6-carbon substrate to support its growth with the production of gas in vitro (Oberg et al., 2021). Gluconate utilization by *Pa. wasatchensis* is achieved by importing the substrate through a gluconate permease, phosphorylation to gluconate-6-phosphate by gluconate kinase, and conversion to ribulose-5-phosphate by 6-phosphogluconate dehydrogenase, which liberates a carbon dioxide molecule (Oberg et al., 2021). In a subsequent research project where sodium gluconate was intentionally added to portions of cheese curd from a 1,500-lb (680-kg) test vat in a controlled Cheddar cheese trial, results showed that significantly more gas was produced in cheese containing added sodium gluconate than in control cheeses with no processing aid added (McMahon et al., 2022). These results indicate that the presence of gluconate in cheese can now be added to the list of confirmed risk factors for unwanted gas production in Cheddar cheese from *Pa. wasatchensis* (or similar NSLAB) along with residual galactose in the cheese, and the use of higher storage temperatures to accelerate cheese ripening.

Addition of sodium gluconate as a processing aid to Cheddar cheese curd has been proposed as a means of preventing calcium lactate crystal formation in cheese by reducing the availability of calcium lactate by forming a soluble calcium-lactate-gluconate complex (Phadungath and Metzger, 2011). In the United States, processing aids can be added during the manufacture of foods to improve processing and may be present in small quantities in the final food (Yerramsetti and Bowser, 2017). Processing aids need to be generally recognized as safe by the US Food and Drug Administration and do not need to be listed as an ingredient when the cheese is packaged. As cheesemaking processes have been shortened to increase efficiency and reduce the cost of manufacture, sodium gluconate can be added while salting the curd as a means of preventing formation of calcium lactate crystals during storage mainly through calcium chelation. Recent observations have shown that this chelation affect imparts a side benefit of allowing the curd to knit together faster so the cheese can be cut and converted into retail packs at an earlier storage time. Prior research has shown that gluconate is not naturally present in milk (Dan et al., 2017; Vandenberghe et al., 2017), and there has not been any published research indicating the natural presence of sodium gluconate in Cheddar cheese.

There are microorganisms that have the ability to produce gluconate from the oxidation of glucose, but the reaction requires oxygen and generates H_2_O_2_, and these organisms are obligate aerobes, as is the case for *Aspergillus* sp., *Gluconobacter* sp., and *Pseudomonas* sp. (Alonso et al., 2015; Bauer et al., 2022; da Silva et al., 2022; Ma et al., 2022). The strict aerobic nature of these organisms would exclude them from being able to grow in Cheddar cheese because of the low redox of the matrix and storage in vacuum bags (Cañete-Rodríguez et al., 2016). In prior research where sodium gluconate was intentionally added to portions of Cheddar cheese curd and measured after pressing and storage, gluconate was only found in cheese when it had been intentionally added (McMahon et al., 2022). The objective of this study was to survey commercial cheeses to determine the use of sodium gluconate as a processing aid in Cheddar cheese.

Fifty-three retail packs of cheese were purchased from grocery stores in Utah representing blocks, shredded cheese, sliced cheeses, and as cracker-sized cuts and sticks, and were labeled as either mild, medium, or sharp indicating different ages. Cheeses samples were measured for gluconate content using enzyme analysis kit K-GATE (Megazyme Inc., Bray, Ireland). For analysis, 5 g of cheese was added to 95 g of distilled water and homogenized using a stomacher at 260 rpm for 4 min, resulting in a 1:20 dilution. The slurry was allowed to stand for 15 min after which the samples were filtered through a coffee filter to remove cheese particles. The filtered samples were tested in duplicate. Following the K-GATE kit instructions, 1,000 μL of distilled water, 200 μL of sample solution, 100 μL of reagent 1 and 2, and 10 μL of reagent 3 were transferred to a 3-mL plastic cuvette. The cuvette was covered with plastic film and inverted 3 times to mix and allowed to incubate at room temperature for 5 min after which the absorption value (**A1**) was measured at 340 nm on a Spectronic 200 spectrometer (Thermo Fisher, Waltham, MA). After A1 measurement, 10 μL of reagent 4 was added to the cuvette, mixed, and incubated at room temperature for 6 min. After the incubation, the absorption value (**A2**) was measured at 340 nm. A blank sample was prepared and treated as described by using 200 μL of distilled water instead of the diluted and filtered cheese samples. The results were converted into percent gluconate according to the kit instructions and adjusted for the 1:20 dilution factor in the cheese. A level of 0.02% or less measured by this assay was considered to be negligible.

For the 14 cheeses labeled as mild, 4 cheeses had gluconate levels ranging from 0.14% to 0.45% (wt/wt) gluconate. Three of these cheeses were shredded and the other was a sliced cheese. Another shredded mild cheese contained 0.06% (wt/wt) gluconate, which suggests a low level of sodium gluconate may have been added, although it is below the level that has been used for preventing calcium lactate crystals. From our previous study in which sodium gluconate was added to curd along with salt (McMahon et al., 2022), it was observed that about 50% to 55% of the added gluconate was lost during pressing. On this basis, the cheese containing 0.06% gluconate would have had only about 0.13% of sodium gluconate added, whereas Phadungath and Metzger (2011) showed that more than 0.5% sodium gluconate was needed to be added to prevent formation of calcium lactate crystals. Sixteen cheeses labeled as medium were analyzed, and 1 shredded cheese contained 0.36% sodium gluconate, 2 cheeses contained low levels of between 0.07% and 0.09% gluconate, whereas the other 13 cheeses were considered to have no added gluconate with gluconate measured at ≤0.02%. Of 16 cheeses labeled as sharp, 2 cheeses show evidence of added sodium gluconate and contained 0.10% to 0.11% gluconate. Both of these were shredded cheeses. The other 6 shredded cheeses had negligible levels of gluconate.

In summary, 7 of the 53 cheeses tested contained gluconate at levels that imply that sodium gluconate was being added to the curd during salting as a way to prevent or delay formation of calcium lactate crystals. These cheeses were mainly converted into shreds and were made by 3 out of 14 companies listed as the manufacturer or distributer on the cheese package.
